# Principles of OCTA and Applications in Clinical Neurology

**DOI:** 10.1007/s11910-018-0911-x

**Published:** 2018-10-18

**Authors:** Adam Wylęgała

**Affiliations:** 1 Ophthalmology Department, Railway Hospital, Katowice Panewnicka 65, 40765 Katowice, Poland; 2 II School of Medicine with the Division of Dentistry, Zabrze Medical University of Silesia, Katowice, Poland

**Keywords:** OCT angiography, OCTA review, Sclerosis multiplex, Neuroscience, OCT-A, Optical coherence tomography angiography,

## Abstract

**Purpose of Review:**

The article reviews the recent findings on the use of optical coherence tomography angiography (OCTA) in neurology*.*

**Recent Findings:**

OCTA is a new addition to the powerful and complementary technology of the OCT. Due to its noninvasiveness, and reproducibility, it is possible to obtain high-resolution 3D images of the vessels of the human eye. As the vessels of the retina with the presence of endothelial cell’s tight junctions resemble the brain vessels, it was hypothesized that the imaging of the retinal vessels might bring insight into brain vessels. OCTA has been effectively used to predict retinal vessel abnormalities in dementia, demyelization, optic disc neuropathies, and inherited degenerative diseases. Most common findings were decrease of vascular density and flow and an increase of avascular zones.

**Summary:**

Although OCTA is a relative new technology, recent studies show that it can be successfully applied in neurology.

## Introduction

The ability of ophthalmologists to detect blood vessels of the fundus was always seen as a great advantage. For over 100 years, various machines to determine blood flow and to visualize blood vessels have been developed. They have differed in their degree of precision, invasiveness, and patient safety [1]. The introduction of optical coherence tomography (OCT) is seen as one of the most fundamental milestones in the history of ophthalmology. It enables 3D imaging of the structures of the eye, e.g., ganglion cell (GCL), macula, optic disc, or cornea, and can measure the size of the optic discs and the thickness of the retinal layers. The submillimeter resolution is easily obtained in histological sections, and the posterior segment penetration is > 1 mm. OCT uses a light source and an interferometer and algorithms to produce images based on the amplitude and the delay of reflected light [2••]. Currently, there are two different technologies utilized by the OCTA devices—spectral domain (SD-OCT) and swept source (SS-OCT). The latter technology is faster and, as it uses longer wavelengths, it provides higher penetration. Moreover, some studies have shown that SS-OCTA has higher sensitivity than SD-OCT [3, 4].

Since 2006, a new software called OCT angiography (OCTA) has been added to OCT devices to enable noninvasive visualization of blood flow. Although it took almost a decade to get this technology on the market [5], OCTA is a useful technology in detection of retinal diseases such as choroidal neovascularization [6], macular malformations in telangiectasias [7], capillary dropouts in diabetic retinopathy [8], perfusion loss in vessels occlusions [7], and changes in flow around the optic disc in glaucoma [9]. In addition to the high reproducibility and repeatability of foveal avascular zone (FAZ) measurements and blood flow in healthy and multiple sclerosis (MS) patients, respectively [2••, 10, 11], the capillary changes in OCTA are also shown to correlate with that of perimetry [12]. The presence of tight endothelial cell junctions in the blood–retinal barrier resembles that of the blood–brain barrier. Although the retinal circulation resembles that of a brain, it lacks autonomic control [13, 14]. As CNS and retinal arterioles share the same embryology and histology [15], measuring the structure and flow within the retinal vessels offers the promise of an objective quantitative and noninvasive technique that in theory corresponds with brain vessel architecture.

**Fig. 1:**
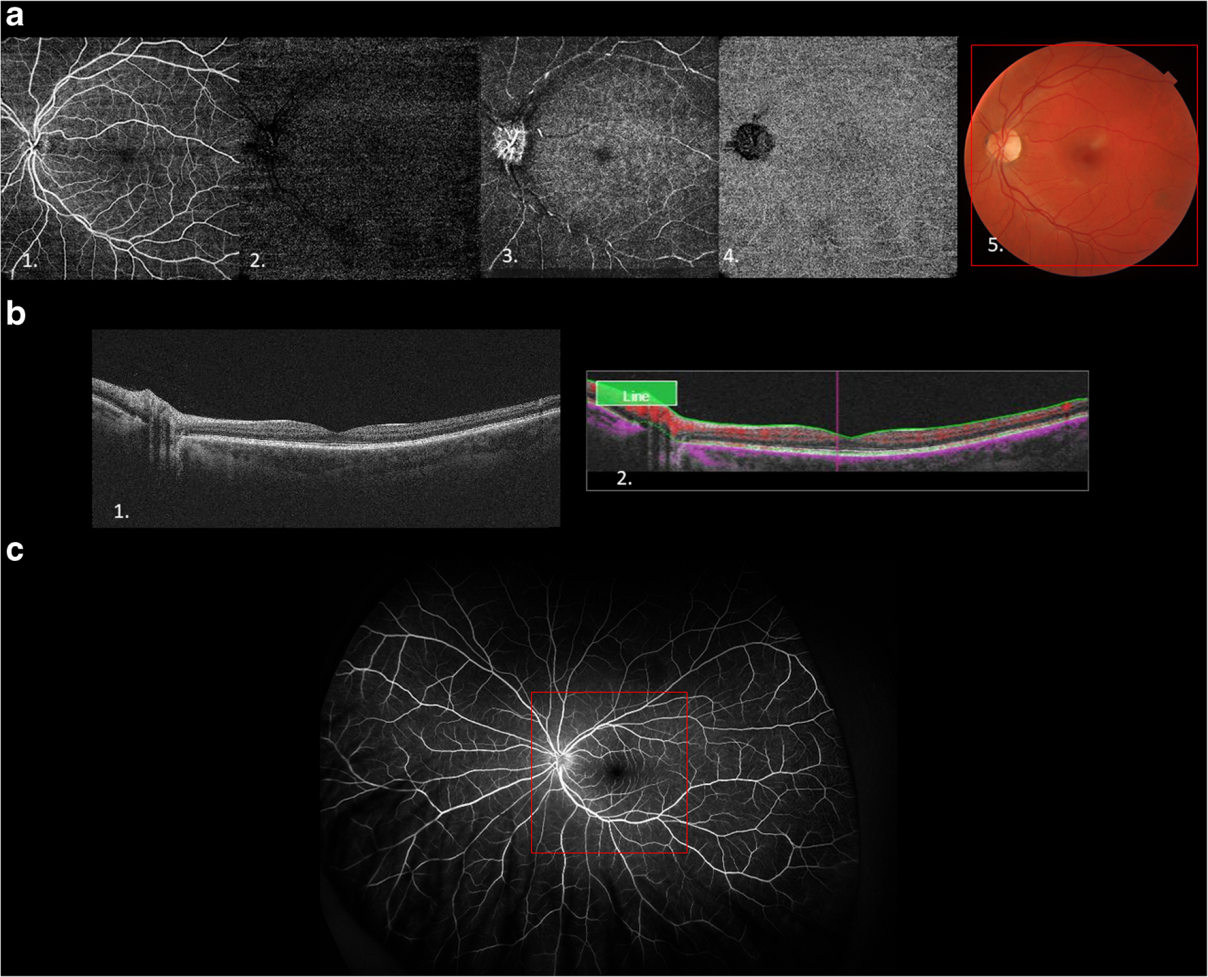
**a** Optical coherence angiography of a healthy retina. 1. Scan of a superficial layers. 2. Intermediate retinal plexus. 3. Deep retinal plexus. 4. Choroid. 5. Color fundus photo with a red frame corresponding to the area on an OCTA image. **b** 1. OCT B-scans of the retina. 2. B-scan with color coded to indicate the blood flow. **c** Images of an ultra-wide field fluorescein angiography, the size of an OCTA image corresponds to the red frame

At present, we understand that the retinal blood supply is organized into four vascular plexuses. The central retinal artery supplies blood to the superficial capillary plexus (SCP), which then anastomoses and creates the intermediate (ICP) and deep capillary plexuses (DCPs). The SCPs are located within the retinal nerve fiber layer (RNFL), ganglion, and inner plexiform layers. While the DCPs are located below, the ICP are located above the inner nuclear layer [14]. Blood vessels are not present in photoreceptors and outer plexiform layers. The fourth retinal plexus is called radial peripapillary capillary plexus (RPC) and runs parallel with the nerve fiber layer axons. RPC contrary to the DCP does not have lobular configuration [1]. Right now, OCTA, as it is coregistered with OCT B-scan, is seen as a method capable to replace fluorescein angiography (FA) in most cases of the retinal and choroidal neovascularizations (Fig. 1). FA is a dye-based technology which visualizes only the SCP due to the blockade of fluorescein from the deeper retinal layers. However, OCTA enables detection of retinal vessels in desired layer [14]. To understand this, let us imagine the difference between a telescope located on earth that offers the astronomer only limited insight into space due to blockage of UV light coming from earth’s atmosphere and a telescope in the outer space with unblocked insight into other galaxies in all light spectrums [16].

### How Does It Work?

OCTA works by collecting multiple cross-sectional scans (B-scans) of the same location and then detecting the differences in motion contrast, amplitude, intensity, or phase. The methods used depend on a particular device. Since motionless objects do not produce any change in a signal contrary to moving objects and the retina and choroid are stationary tissues, the differences in values are believed to be coming from the only moving particle within them which is blood. Erythrocytes, due to their biconcave structure, reflect light. Such scanning is then performed in different axes to create a 3D image [17, 18].

### What Can Be Measured in OCTA?

The primary function of this technology is to visualize the architecture of blood vessels without the need to inject the dye. It is important to know that the image that is being created, unlike in FA, is just a result of a mathematical calculation of the computer. This can be useful in calculating various parameters including blood flow [19], vessel density, size of a vascularized and FAZ [20], size of perfusion [21], fractal analysis, or even measurement of complexity of a mathematical tree [22]. Furthermore, the descriptive morphologies of the vessel architecture like diameter, length, or number of branches can also be calculated [22].

## Methods

A literature search was conducted including all articles published up to September 2018. A PubMed, Web of Science, Google Scholar, and Mendeley search with reference cross matching was used to identify all relevant articles pertaining to OCT angiography, dementia, neurology, brain, mild cognitive decline, neuritis, multiple sclerosis, and neuropathy.

### Dementia

Primary risk factors of neurodegenerative disorders are advanced age and cardiovascular disease. With the increase in life expectancy, new methods of screening are needed [23]. At the level of cerebral microvasculature, there is a growing evidence of age-related drop in capillary numbers and density observed in humans [24] and in rodents [25, 26]. Diagnostic methods for detecting and defining neurodegenerative syndrome routinely used by neurologists such as CSF assessment, PET, and MRI are either limited by their invasive nature or high costs [27]. Alzheimer’s disease (AD) is considered the most common type of dementia. Common ophthalmologic signs and symptoms of AD are visual field changes and decreased visual acuity [28]. Pathologically, it is characterized by deposition of plaques and tangles that consist of amyloid and tau protein, respectively. It is estimated that retinal microvascular changes such as microaneurysms, soft or hard exudates, retinal hemorrhages, macular edema, intraretinal microvascular abnormalities, venous beading, new vessels, vitreous hemorrhage, or disc swelling are associated with cognitive decline [29]. Moreover, other retinal alterations such as changes in the thicknesses of macular RNFL are associated with early changes in Alzheimer-type dementia [30]. Although other studies showed a decrease in ganglion cell layer (GCL) [31], the changes in RNFL and inner plexiform layer [32] are associated with the neocortical Aβ accumulation, while GCL is not [33]. A recent study evaluated OCTA findings in AD. The authors described enlarged FAZ measured by the OCTA device and decreased retinal vascular density and choroidal thickness which correlated with a decrease on Mini Mental State Examination. They attribute these changes to decreased angiogenesis caused by binding of vascular endothelial growth factor (VEGF) to Aβ plaques [34•]. In a comparative study on twins, it was observed that a twin with AD had larger FAZ in SCP and thinner choroid compared with the healthy sibling [35]. Similarly, in a study by Jiang et al., AD patients had reduced density of both SCP and DCP in comparison with controls, whereas patients with mild cognitive impairment had reduced density only in DCP and in the superior nasal quadrant [36]. Thus, as the blood from the central retinal artery supplies firstly SCP and then DCP, reduced SCP density can lead to decreased blood flow through the outer layers of retina which may lead to continual loss of ganglion cell axons [37].

In migraine, mainly peripapillary RNFL thickness is reduced, especially in patients with aura [38]. One study demonstrated reduced parafoveal SCP density and superior RPC density in patients with migraine with aura compared with migraine without aura and healthy controls. Furthermore, patients with aura had enlarged FAZ. However, the authors speculate that this is probably associated with increased risk of retinovascular occlusion, normal tension glaucoma, and ischemic optic neuropathy among patients with migraine. The authors did not observe significant changes between migraine without aura and healthy controls [39•].

The article by Nelis et al. describes changes in OCTA observed in 21 eyes of 11 patients with cerebral autosomal dominant arteriopathy with subcortical infarcts and leukoencephalopathy (CADASIL). The patients showed decreased vessel density in DCP which the authors associate with Notch3 mutation in pericytes. However, only 9 patients included had confirmed Notch3 mutations [15]. Notably, this corresponds to a report of OCT findings by Fang et al. where the inner arterial diameter and arterial to venous ratio of the inner and outer diameters correspond negatively to a number of small infarcts in the 7T MRI [40].

Falavarjani et al. evaluated optic nerve microvasculature in 21 eyes of patients with optic disc edema, pseudoedema, and atrophy and compared them to 12 heathy eyes. They observed decrease in the RPC which was more outstanding in areas with sectorial optic atrophy. The capillaries of the patients with disc edema were dilated and tortuous. The mean capillary density was significantly decreased in eyes with disc atrophy. Moreover, the RPC vessel density correlates with the NFL thickness. The authors hypothesize that reduced metabolic need is caused by reduced number of nerve fibers; however, it had not been the subject of the study [41].

### Optic Disc Ischemic Neuropathies

The role of OCTA in detecting vascular optic nerve abnormalities is even more promising. Ischemic injury to the optic disc is a clinical syndrome known as anterior ischemic optic neuropathy (AION). This syndrome can be divided into arteritic (AAION) and non-arteritic (NAION). AAION is a focal manifestation of giant cell arteritis, a systemic inflammatory disorder, and the perfusion defect is located at the level of posterior ciliary artery. In the NAION form, the perfusion defect is at the distal end of the posterior ciliary circulation at the level of paraoptic branches [42]. Fard et al. using OCTA observed a 2.2% loss of RPC compared with the healthy group [43]. Furthermore, Ling et al. using OCTA showed a 7.23% increase in the size of nonvascular tissue in 21 eyes compared with the controls’ eyes with NAION [44]. Patients with NAION have unilaterally decreased RPC density, whereas glaucomatous patients were bilateral [45]. Moreover, a positive correlation between decrease of RPC, RNFL [46], and GCC [46] was observed. Although, at present, OCTA is not helpful in distinguishing AION from NAION [42], it is reasonable to consider the possibility that the changes located proximal to the division of posterior ciliary arteries into paraoptic and choroidal branches affect RPC in both disorders.

### Demyelinating Disease

Multiple sclerosis (MS) is a chronic demyelinating inflammatory disease. Autopsy revealed that 99% of patients have had signs of demyelination within the optic nerve [47]. Optic neuritis is a primary manifestation in 25% of patients and can occur in 50% of the patients during the course of the disease [48••]. Although several articles concerning OCTA of patients with MS appeared in the literature, one of the earlier studies showed that optic nerve head (ONH) flow measured within the whole optic nerve head with an experimental device in patients with optic neuritis was reduced by 12.5% compared to controls. Furthermore, the flow index was also lower in patients without optic neuritis compared with controls. Moreover, 21% of MS patients with correct visual acuity showed abnormal flow [11].

Spain et al. showed reduced ONH perfusion by OCTA in patients with MS, particularly those with a history of optic neuritis. Although combining OCT angiography with other OCT parameters increased the diagnostic accuracy of detecting MS in eyes with optic neuritis, there was no significant correlation among flow index and GCC and RNFL [48••]. They used the same experimental procedure as Wang et al. [11]. One relatively large OCTA study documented that lowered vessel densities in both SCP and DCP in the perifoveal region are correlated with decreased volume of corresponding retinal layers compared with the healthy controls. Furthermore, there was an increase in choriocapillaries density which the authors linked to the inflammation within the last 24 months [49••].

Overall, the literature indicates that MS leads to decreased retinal ganglion cells and RNFL thickness measured in OCT, which results in lower metabolic activity and rarefication of the vascular plexus. Some authors advocate the presence of autoregulatory mechanism or alterations in endothelium that were observed in MS brains. The authors attribute the lack of changes in the parafoveal regions among the groups to the lack of macular RNFL and the fact that macula is supplied by the choroid and the range of autoregulation is much bigger than for the regions supplied by the retinal vessels [11]. However, they did not differentiated retinal vascular plexus but measured flow index in the whole retina.

Neuromyelitis spectrum disorder (NMOSD) with AQP4-IgG is characterized by the presence of AQP4-IgG and one of the clinical characteristic: optic neuritis, acute myelitis, acute brainstem syndrome, area postrema syndrome, symptomatic cerebral syndrome with NMOSD brain lesions, symptomatic narcolepsy, or acute diencephalic clinical syndrome with NMOSD-typical diencephalic MRI lesions [50]. There are some reports on the functionality of OCTA in NMOSD. A recent study assessed peripapillary and parafoveal vascular network in aquaporin-4 antibody positive NMOSD. The study team evaluated 108 eyes of 67 patients divided into two groups based on the history of optic neuritis. Moreover, 66 eyes of 33 healthy participants were added into study as a control. RPC vessel density was significantly reduced compared with both controls (15.7%) and non-neuritis patients (13.5%). The parafoveal density was decreased to 3.2% and 5.9% in neuritis patients compared with non-neuritis patients and controls, respectively. In addition, a positive correlation between OCT parameters which are RNFL, GCL thickness, and OCTA capillary density in both measured regions was observed [51••]. In the retina, the Müller glial cells expressing aquaporin-4 which regulate blood flow [52] and support neurons may be directly targeted by aquaporin-4 antibody [51••]; the loss of which may lead to thinning of the retinal layers and reduced vascular density [53]. The authors concluded that vascular changes occur prior to the development of optic neuritis and advocate OCTA as a future tool for detecting subclinical vasculopathy in NMOSD patients without optic neuritis [51••]. Same conclusions were drawn in that the decrease in capillary density of both SCP and DCP observed in OCTA correlates and partially contributes to neuroaxonal thinning of the retina [54].

### Optic Pathway Pathologies

Leber hereditary optic neuropathy (LHON) is a hereditary mitochondriopathy that causes painless vision loss, typically bilateral, that often affects young males. Its signs include transient telangiectasia seen in OCTA [55–57], swelling of the RNFL (pseudoedema) in OCT, and absence of leakage in FA [57, 58]. In a OCTA study of 22 patients with LHON divided into four groups (unaffected carriers of the mitochondrial DNA mutation, symptomatic patients in the early subacute phase, late subacute, and chronic), a significant drop in RPC density (19.06%) was first observed in the temporal regions of early subacute patients compared with the unaffected carriers. Whereas the late subacute patients had, in general, a decreased temporal RPC density of 24.27% and a whole RPC density of 8.99%, and chronic patients had a decreased temporal RPC density of 36.09% and a whole RPC density of 24.65%. While in the chronic patients, decreased whole RPC density was observed, the RPC density of unaffected carriers increased in temporal regions together with RNFL thickness compared with controls. The authors explain this fact by swelling of the axons in both unaffected and acute stage patients as a compensatory aggregation of mitochondria or due to the failure of axonal transport that leads to microangiopathy. As small optic discs are risk factors in LHON, another explanation is that the insufficient vascular supply causes axonal swelling leading to compartment syndrome and to degeneration of ganglion cells [59–61]. Based on the current literature, it is impossible to determine whether the microangiopathy is causing or correlates with the RNFL swelling.

Later, during the course of disease, this swelling turns into atrophy. Secondly, the vascular decrease occurs faster than a decrease in RNFL thickness which the authors attribute to the slower resolution of swelling [60•]. Another OCTA study showed a 9.1% and 9.4% decrease in SCP and DCP, respectively, compared to healthy subjects. This study also observed higher decrease in the inferior and temporal regions of the disc where the papillomacular bundle is located. However, in contrary to decrease in SCP that correlates with decrease in visual loss, the authors observed no correlation between the visual loss and OCT structural damages [55].

Another study reported the use of OCTA in patient with a Wolfram syndrome, a rare neurodegenerative disease including diabetes insipidus, diabetes mellitus, deafness, and optic neuropathy. This entity is associated with a defect in transmembrane protein that maintains calcium homeostasis. The OCTA showed reduced vessels in RPC and SCP that corresponded with the OCT-measured RNFL thinning. Morphologically, the vessels were telangiectatic and tortuous. The authors compared the findings with the OCTA results of LHON and dominant optic neuropathy patients and speculate on the possible mitochondrial involvement [62]. Parrozzani et al. advocate that retinal vascular remodeling is a consequence of axonal degeneration.

In an OCTA study on 26 patients with posterior optic pathway gliomas involving the chiasma, retrochiasmal, or both, they observed reduction of macular perfusion in DCP, whereas SCP was not affected by this entity. Furthermore, reduction in OCT-measured RNFL is correlated with the decrease in peripapillary perfusion. Therefore, the authors demonstrated that the decrease of retinal perfusion is secondary to reduction of retinal neurons which leads to reduced metabolism. They suggest a pivotal role of Müller cells in this feedback mechanism [63••].

## Discussion

OCTA opens unique opportunities and already showed great potential in visualizing the blood vessels. Even early studies using fundus photography showed association between retinal vasculature and a risk of stroke [64, 65], or cognitive ability [66, 67]. OCTA, however, is not free from shortcomings—the biggest being the presence of artifacts coming from the motion of the eye or from the other vessels. Not all light is transmitted from the erythrocytes, e.g., melanosomes of the choroid backscatter light so that the choroidal vessels are imagined dark [68]. Despite constant upgrades in software, the motion artifacts are still limiting the use of the instrument in older patients and children with poor cooperation as they have to fixate their eyes for some time [22]. Moreover, presence of epiretinal lesions such as myelinated fibers may also obscure the images [69]. Studies conducted in animal models show that injecting contrast agents that are more symmetrically shaped than the erythrocytes allows us to observe images with less artifacts as some of the artifacts are inherent to the method of detecting erythrocytes [68].

OCTA is an emerging technology with still many limitations, requiring careful use and interpretation. The literature on the topic is of mediocre quality with mostly small, single-center retrospective studies [43–46, 63••, 70–72]. Some authors have calculated parameters based on several instruments which can lead to inaccurate results [43]. Others had to export the images to another program which could also reduce the quality of the results [15]. The changes demonstrated in the articles like enlargement of FAZ or decrease in capillary plexuses density are unspecific and were observed in many other conditions like diabetic retinopathy [73], vessels occlusions [74], or post-surgery [75]. Likewise, there is no body of literature addressing the relation of OCTA-imaged vessels and treatment in neurological research. Last but not least, the manufacturers use different algorithms for detecting retinal motion and segmenting retinal layers making it difficult to compare the results between the studies [17, 76]. In addition, in all studies, only perimacular and papillary regions were assessed. To conclude now, it is not possible to make a diagnosis based on the OCTA images or blood flow.

Calculation of blood flow may lead to better understanding of pathology and facilitate diagnosis of many neuro-ophthalmic diseases. However, it is still unknown how reliable are the results obtained by the machines because, firstly, the algorithms are sensitive to a certain speed—below or above which the flow will not be detected nor the vessels visualized—and, secondly, the accuracy of the flow speed depends on OCT scan quality—images that are out of focus, full of artifacts coming from optical opacities, and motion artifacts will produce false results [77]. Whether flow indexes will enter the clinical practice is also unknown. Another controversy exists whether the observed changes in the retinal vasculature directly reflect the brain microvasculature or are secondary to the vascular risk factors, e.g., aging [76]. A major methodological challenge exists as, at the time of writing this review, the OCTA technology is still under rapid development.

Alternative techniques currently used in vessel visualization:

Currently, a few alternatives exist to OCTA.

Currently, many manufacturers sell OCT devices with OCTA functionality. Moreover, many prototypes [78–80] with much higher speed and penetration are used in research. However, OCTA is not the only technology used in detection of vessels and measuring flow. Adaptive optics is a tool of correcting scanning laser ophthalmoscopy that allows to observe structures as small as single photoreceptors. However, this method is time consuming and produces only small field of view [1, 81, 82].

While Doppler OCT yields flow in microliter per minute, OCTA can provide date in arbitrary units only; however, it can only be used in larger vessels and perpendicular to the probe [21].

A current gold standard technology—FA with fundus photo— is limited by its 2D size and its inability to visualize a particular layer as it creates a merged image of only microscopical vessels. FA is associated with some side effects, the most severe, however, very rare being anaphylactic shock. OCTA has also much higher resolution than FA which makes it possible to detect small areas of retinal non-perfusion that can resemble cerebral microinfarctions [83]. Furthermore, there is no need to inject the dye and, hence, the possibility to reproduce the examination as many times as the examiner wants. OCTA is more sensitive than dye-based techniques in detecting small perifoveal capillaries and radial peripapillary capillaries [84]. As it is not dependent on dye reflectance, it can produce more sharp images than FA, in which borders cannot be seen with such contrast due to leakage [85]. However, FA is well established and many diseases have particular features seen only in FA. Therefore, performing FA may be crucial for diagnosis.

## Conclusion

Advancements in noninvasive techniques for measurement blood flow are likely to change many medical fields including neurology. More prospective studies are needed with the use of medications to observe changes in vascular parameters and architectures to validate initial reports. Another field of future studies should be the use of OCTA in stroke, neurodegenerative psychiatric disorders such as schizophrenia, and determination of retinal blood flow in other pathologies like Uhthoff’s phenomenon [86, 87].
